# Seeking Relevant Biomarkers in Common Variable Immunodeficiency

**DOI:** 10.3389/fimmu.2022.857050

**Published:** 2022-03-11

**Authors:** Hsi-en Ho, Charlotte Cunningham-Rundles

**Affiliations:** ^1^ Department of Medicine, Icahn School of Medicine at Mount Sinai, New York, NY, United States; ^2^ Department of Pediatrics, Icahn School of Medicine at Mount Sinai, New York, NY, United States

**Keywords:** common variable immunodeficiency, primary immunodeficiency, biomarkers, complications, pathogenesis, genes, environment

## Abstract

Common variable immunodeficiency (CVID) is the most common symptomatic form of primary immunodeficiency. More than 50% of patients in some series suffer from autoimmune or inflammatory complications (the “CVID+” phenotype), and these are not adequately addressed by current treatments. Despite major advancements in genetics, the pathogenesis of the CVID+ phenotype has remained unexplained for most patients, necessitating the need for relevant biomarkers in both the clinic and research settings. In the clinics, reduced isotype-switched memory B cells (≤ 0.55% of B cells) and reduced T cells (CD4) can be utilized to identify those with increased complication risks. Additionally, condition-specific markers have also been suggested for lymphoma (normal or elevated IgM) and progressive interstitial lung disease (increased BAFF, normal or elevated IgM). Additional biomarkers have provided insights into disease pathogenesis, demonstrating wider systemic inflammation (increased LBP, sCD14, and sCD25; expanded ILC3), mucosal defects (increased zonulin, I-FABP), and perhaps reduced anti-inflammatory capability (reduced HDL) in CVID. Most recently, efforts have revealed elevated circulating bioactive bacterial DNA levels – marking microbial translocation and potentially linking the causation of multiple inflammatory changes previously observed in CVID. The implementation of high throughput profiling techniques may accelerate the search of relevant biomarker profiles in CVID and lead to better clinical risk stratification, revealing disease insights, and identifying potential therapeutic targets.

## Introduction

Common variable immunodeficiency (CVID) is one of the most common symptomatic primary immunodeficiency disorders, with an estimated prevalence of 1:50,000 to 1:25,000 (1–3). While most subjects are adults at the time of diagnosis, diagnostic delay of many years is prevalent. After establishing the diagnosis, the immune defects require various and lifelong therapies. Due to the loss of antibody function, most patients experience intermittent infections. With the emergence of immune globulin therapy as the mainstay of treatment in the past 3 decades, infection frequencies and related hospitalizations have been reduced (4). However, it has also become clear that CVID has two distinct phenotypes: one group with recurrent infections that can be addressed by adequate immunoglobulin replacement, and a second group with additional autoimmune/inflammatory complications (“CVID+”) as the main manifestation (2). These non-infectious complications are usually evident at initial presentations, though they may also appear subsequently. They include progressive interstitial lung diseases, autoimmunity, gastrointestinal inflammatory diseases, granulomas, liver diseases, excessive lymphoid hyperplasia, and the development of cancers, especially lymphoma (2, 5). These manifestations of immune dysregulations have become the major challenge since they are not ameliorated by standard immune globulin replacement therapy and the pathogenesis remains poorly understood (2, 6).

Cohort and registry data have provided insights to the clinical determinants of health outcomes in CVID, as well as the CVID+ phenotype. Data from the Mount Sinai cohort showed that non-infectious complications led to both increased morbidity and mortality in CVID (2). ESID Registry data for 2700 patients demonstrated that increased mortality was additionally associated with older age at diagnosis, later onset of symptoms, diagnostic delay, and parental consanguinity, but notably, not immunoglobulin replacement dosage (7). USIDNET Registry data for 990 patients showed that while some CVID+ patients may only have one non-infectious manifestation, the vast majority in this group suffered from multiple inflammatory complications concurrently (8). For instance, lymphoproliferative disease and granulomas were significantly associated with hematologic autoimmunity, interstitial lung disease, and hepatic/gastrointestinal diseases. This insight demonstrated the burden of disease for patients and additionally suggested a shared pathogenesis underlying these varied conditions.

With the large-scale application of genetic sequencing in CVID, it was widely hoped that the rapid identification of specific genes responsible for the CVID+ phenotype would drive our understanding of the disease, and to an extent, this has occurred. In 10-30% of CVID patients, pathologic monogenic defects can be identified (9–11). These defects are broadly categorized into 1) genes implicated in various stages of B cell activation, survival, or maturation to the plasma cell stage and 2) immune-regulatory genes, with autoimmunity and inflammation (CVID+) being more characteristic of the latter group. In a study of 571 CVID patients from three cohorts (New York, Sweden, and Iran), genes considered causative were identified in 31% (New York), 36% (Sweden), and 54% (Iran) of participants (11). For CVID+ patients, the yield of genetic sequencing was somewhat higher (35-36.9% in New York and Sweden). However, the majority of CVID patients, including those with numerous complications, did not have an identified causative genetic mutation (63.1-65% of CVID+ patients in New York and Sweden; **Table 1**). Another relevant question is whether the clinical conditions suggest which mutations might be present. When examining patients with mutations in the same gene in the more similar New York and Swedish cohorts, it was clear that clinical conditions would not necessarily be a useful indicator of the underlying genetic defect. Overall, genetic causes of at least ~70% of CVID patients remain unidentified to date, including those with complications. Presently, it is clear that other genetic changes are likely operative, and much more work remains to be done through a genetic approach.

**Table 1:** Monogenic defects identified in 3 CVID cohorts by phenotype (infections only vs. complications) (11).

	Gene identified, *n* (%)	No gene identified, *n* (%)
**United States, *n* = 234**		
Infections only, *n* = 91	23 (25)	68 (75)
Complications,* n = 143	50 (35)	93 (65)
**Sweden, *n* =113**		
Infections only, *n* = 40	11 (27.5)	29 (72.5)
Complications,* *n* = 73	27 (36.9)	46 (63)
**Iran, *n* = 188**		
Infections only, *n* = 80	43 (53.7)	37 (46.2)
Complications,* n = 108	62 (57)	46 (42.5)

*CVID complications include lymphocytic and/or granulomatous infiltrations, lymphadenopathy, splenomegaly, autoimmunity, and noninfectious enteropathy.

While genetic research efforts continue, and will certainly lead to additional discoveries, dissecting the CVID phenotype, especially the autoimmune/inflammatory conditions, continues to be the focus of major research activities. In particular, there have been considerable efforts to find pertinent biomarkers that may be useful to 1) identify CVID patients at particular risk for developing complications, 2) provide clues to the shared pathogenic pathways, and 3) potentially offer insights to new therapeutic approaches. Here, we distill and tabulate the evolution of these biological markers in the past few decades and discuss current work on the identification of relevant biomarkers in CVID (**Table 2**).

**Table 2:** Relevant clinical and laboratory biomarkers in CVID.

Clinical risk markers	Laboratory Biomarkers	Laboratory Biomarkers Notes
Older age at presentation	Very low isotype switched memory B cells	≤ 0.55% of B cells: risk factor for granulomas, splenomegaly and autoimmunity
Later diagnosis of CVID	Increased CD21^low^ anergic B cells	Linked to splenomegaly and autoimmune cytopenia
Diagnostic delay	Increased transitional B cells	Linked to lymphadenopathy
Consanguinity	Normal or increased serum IgM	Association with lymphoma and progressive ILD
Selected inflammatory complications	Low serum soluble BCMA	Distinct in CVID when compared to IgG deficiency and selective IgA deficiency
Lymphoma	Interferon mRNA signature	Distinguish CVID+ from other CVID patients and non-CVID healthy controls
Granulomatous and/or lymphocytic lung disease	Expanded innate lymphoid cells	Potential source of IFN-γ in CVID+, most prominent in those with autoimmunity and/or lymphoproliferation
Splenomegaly	Increased sCD14 in blood	Highest levels in CVID+
Autoimmunity	Increased sCD25 in blood	Highest levels in CVID+
	Increased serum BAFF and APRIL	Serum BAFF is inversely related to isotype switched memory B cells; may mark patients with progressive ILD.
	Increased LBP	Associated with increased sCD14 in blood, may be an indirect biomarker for microbial translocation in CVID.
	Low serum HDL	Reduced HDL levels and impaired function linked to systemic immune activation in CVID
	Increased serum TMAO	Correlated with selective inflammatory cytokines in CVID
	Low or absent IgA	Linked to gastrointestinal inflammation and intraepithelial lymphocytosis
	Increased serum I-FABP	Widely present in CVID regardless of overt enteropathy
	Increased serum zonulin	Widely present in CVID regardless of overt enteropathy
	High serum levels of gut commensal bacterial DNA	Marked the CVID+ phenotype and those with low isotype-switched memory B cells (<2% of total B cells); high levels marked higher serum IFN-γ; bioactive in *ex vivo* studies of CVID circulating cells.

APRIL, a proliferation inducing ligand; BAFF, B cell activating factor; BCMA, B cell maturation antigen; I-FABP, intestinal fatty-acid binding protein; ILD, interstitial lung disease; HDL, high-density lipoprotein; LBP, lipopolysaccharide-binding protein; sCD14, soluble CD14; sCD25, soluble CD25; TMAO, trimethylamine N-oxide.

## Circulating Biomarkers

### B and T Cell Phenotypes

Circulating lymphocyte markers were the first extensively studied biomarkers in CVID, and they were quite successful in identifying patients at particular risks for developing non-infectious manifestations. Overall, patients with severely reduced peripheral isotype-switched memory B cells, increased CD21^low^ B cells, and reduced T cells (especially naïve and regulatory T cells) were more likely to have these complications (12–15). In the B cell compartment, switched memory B cell phenotypes have been the most widely utilized markers. In particular, decreased switched memory B cells (cut-off: ≤ 0.55% of B cells) was an independent risk factor for granulomas, splenomegaly, and autoimmunity (15). Similarly, a cut-off of <2% switched memory B cells was identified as a risk factor for granulomas and splenomegaly in another study (12). On the other hand, the expansion of CD21^low^ B cells (>10%) had been linked to splenomegaly and autoimmune cytopenia (12, 16). Lastly, the expansion of transitional B cells (>9%) was linked to lymphadenopathy (12). Interesting, we had also observed an association between sex and distinct B cell profiles (15). Specifically, female CVID patients had significantly more switched memory B cells and higher serum IgM levels than males. At present time, however, it is not known whether this B cell differential is linked to the higher incidence of B cell lymphoma observed in female CVID patients (2, 5). In the T cell compartment, reduced peripheral T cells have been observed in CVID+ cases in most cohorts. In rare cases, severe CD4 T cell lymphopenia (<200 cu/mm) is observed, and it is linked to risk for opportunistic infections (14). These cellular biomarkers are now commonly utilized at early encounters to allow better initial classifications and provide some level of risk assessment.

### Cytokines

Many research studies over the years have also examined cytokine markers in CVID, with the goals of illuminating the immune defect and providing additional measures in following complex cases. Studies of cytokines have been reviewed in-depth previously (17). However, results between studies can be difficult to interpret due to differences in patient selection, cell types examined, and experimental conditions. One of the first cytokine abnormalities noted was a lack of IL-2, potentially related to the observed T cell defects (18, 19). This had led to clinical trials of IL-2 in CVID and resulted in some correction of T cell proliferation defects (20). Studies also suggested increased TNF-α, both in serum and in peripheral blood mononuclear cell (PBMC) stimulation studies (21). Additionally, a polymorphism in TNF (the TNF+488A allele) had been noted in a CVID subset characterized by granulomas, splenomegaly, lymphopenia, reduced CD4+CD45RA+ T cells, and expanded CD8+CD57+ lymphocytes (22). Due to these suggestions, anti-TNF-α therapies was utilized in case reports of severe enteropathy and granulomatous disease, suggesting potential utility in specific complications (23, 24). However, evidence remains at the case report level and careful consideration of secondary infection risks with anti-TNF-α therapies is required. IFN-γ abnormalities in CVID had differed between cell types and patient subgroups (25, 26). However, in a larger cohort, we had found abundant serum IFN-γ in CVID+ patients, along with an IFN mRNA signature that distinguished CVID+ from CVID patients and healthy volunteers (further discussed below) (27, 28). A number of other cytokines had also been examined in CVID, though the results often varied between studies: IL-4 (elevated serum levels in most studies, but reduced in some) (29, 30), IL-5 (potentially reduced) (31), IL-6 (increased production in some studies) (32, 33), IL-7 (varied) (34), IL-10 (varied) (35, 36), and IL-12 (varied) (37, 38).

### B Cell-Activating Factor, A Proliferation Inducing Ligand, B Cell Maturation Antigen

Both BAFF and APRIL belong to the TNF family and are clearly elevated in CVID serum (39). BAFF is known to drive immature B cells, and elevated BAFF/APRIL system is associated with systemic autoimmune disease, such as systemic lupus erythematosus (40). Thus, there has been substantial interest in their roles in the CVID+ phenotype. Among CVID patients, serum BAFF levels are inversed related to peripheral B cell numbers (41) and switched memory B cells (unpublished observation), suggesting a role in driving B cell production and maturation in this condition. In our initial studies (n=77), we did not find a clear relationship between BAFF/APRIL and clinical parameters (autoimmunity, lymphadenopathy, splenomegaly) or TACI mutations (39). However, we subsequently found that elevated serum BAFF marked CVID patients with progressive interstitial lung disease (ILD) from those with stable ILD, other CVID patients, and healthy volunteers (42). In addition, we detected a relative abundance of BAFF in the lung tissues of progressive vs. stable CVID ILD. Together, these results indicate that elevated BAFF (in blood and lungs) could be a biomarker that distinguishes CVID patients with progressive ILD.

In contrast, serum B cell maturation antigen (BCMA), a TNF receptor expressed largely on the surface of plasma cells and plasmablasts and promotes survival of these cells, appears to be reduced in CVID (43). In this study, we found that serum soluble BCMA was significantly reduced in CVID when compared to those with milder forms of antibody defects, including IgG deficiency and selective IgA deficiency. Serum soluble BCMA levels positively correlated with plasma cell counts in the gastrointestinal mucosa.

### Serum IgM

The relative abundance of serum IgM is another interesting biomarker in two specific CVID-associated complications – namely, lymphoma and ILD. The connection to lymphoma was first suggested in a landmark study from the ESID registry, where CVID patients who developed lymphoma were found to have higher baseline serum IgM compared to other CVID patients (44). This was also recently validated in our study of 45 lymphoma cases (B cell type: n=44) amongst a total of 647 CVID patients (45). Here, we found that patients with lymphoma had a median baseline serum IgM of 74.5 mg/dL (range 4-830), significantly higher than the rest of the cohort (median 32.2 mg/dL, range 0-645, P=0.009). In a separate study examining CVID-associated ILD, we had found that the retention or increase of serum IgM was associated with progressive lung disease (42). This observation paralleled BAFF-driven B cell hyperplasia, and was associated with increased lymphoid infiltrates and germinal center formation in the lung tissues, and suggested serum IgM as a relevant non-invasive biomarker in CVID patients with ILD.

### LPS Binding Protein, Soluble CD14, Soluble CD25, and HDL

While much of the research efforts in CVID have focused on adaptive immune abnormalities, there has also been an increased appreciation of a wider systemic immune activation in CVID. Both LBP and sCD14 are considered to be acute phase proteins, typically released by hepatocytes/intestinal cells and monocytes/macrophages, respectively, in response to microbial stimuli (46–48). Multiple studies had previously reported elevated circulating LBP and sCD14 in CVID compared to healthy controls (49, 50). This was also confirmed in a larger CVID cohort (n=76), where we found that the highest sCD14 levels were amongst CVID+ patients, supporting innate immune activation as an important hallmark of CVID, especially for those with non-infectious manifestations (28). Here, we also observed a positive trend between serum sCD14 and LBP(Spearman r = 0.21, P = 0.0735). Others had additionally reported a positive correlation between sCD14 and sCD25, typically produced by activated T cells (though not exclusively), in CVID, with the highest levels of both markers observed in CVID+ (50, 51). Another quite original and potentially relevant biomarker in CVID is high-density lipoprotein (HDL). This was examined due to its known anti-inflammatory properties, for instance, in reducing toll-like receptor (TLR)-induced inflammation in macrophages (52). In a large CVID cohort (n=102), plasma HDL was reduced compared to healthy controls, especially in those with non-infectious complications (53). In addition, plasma HDL was inverse correlated with inflammatory markers CRP and sCD25 in this cohort, suggesting a link between impaired HDL levels/function and immune activation in CVID.

## Systems Profiling

### mRNA Transcriptional Profiling and Mass Cytometry

In recent years, high throughput molecular and cellular profiling techniques have been increasingly utilized to identify biomarkers in complex and heterogeneous disorders. Taking this open-ended approach, we had first conducted whole blood mRNA profiling in a large CVID cohort (n=91) (54). Here, we found a marked up-regulation of IFN responsive genes that distinguished CVID+ patients from other CVID patients and control participants. In parallel, we also found a pronounced down-regulation of transcripts related to the B cell, plasma cell, and T cell modules that characterized CVID+ patients. Further investigation identified type II IFN (IFN-γ) signature as a heightened cytokine pathway in CVID+. To trace the cellular origin of IFN-γ in CVID, we subsequently employed mass cytometry and found that the circulating IFN-γ+ cells had markers of innate lymphoid cell type 3 (ILC3; lineage negative, CD127+, CD161+, T-bet+, and retinoid acid-related protein receptor-γ+) (27). These IFN-γ+ ILCs were also positive for IL-17A and IL22. They were not only expanded in the peripheral blood of CVID+ patients (mean 3.7% of PBMCs) but were also found in the end organs of disease (gastrointestinal and lung tissues). In support of this finding, increased ILC3 cells were similarly identified in another CVID cohort, most prominently in those with autoimmune and/or lymphoproliferative complications – this was accompanied by a relative loss of ILC2 cells in this study (55).

### Serum Proteomics

While earlier studies have focused on measuring individual cytokines in the circulation and in experimental settings, the availability of new protein multiplex platforms have provided another open-ended approach to profile immune-related protein biomarkers in CVID. In a recent study that combined a serum proximity extension assay and machine learning, three main proteins were found to be associated with the CVID+ phenotype: IL-10, IL-12 receptor subunit beta 1 (IL12RB1), and CD83 (56). Additionally, mast cell immunoglobulin-like receptor 1 (MILR1) and leukocyte immunoglobulin-like receptor subfamily B member 4 (LILRB4) also distinguished the CVID+ patients in the machine learning training cohort (though not replicated in a separate testing cohort). Confirming prior studies, dysregulated IFN-γ responsive chemokines, CXCL9, 10, and 11 were also noted in CVID+, again confirming a Th1 skewing in these patients.

### Spectrochemical Analysis of Blood

Another open-ended approach is the application of Fourier-transform infrared (FTIR) spectroscopy, a high-resolution spectroscopy method, to detect discriminatory molecular and structural changes in CVID (57). In a proof-of-concept study, this technique was capable of segregating CVID patients from healthy participants based on serum or plasma samples with high sensitivity and specificity. In addition, the plasma ‘fingerprint’ derived was able to segregate CVID+ from other CVID patients with high specificity, albeit with much lower sensitivity. Here, biomarker analysis between CVID and healthy participants revealed discriminating signals in regions associated with nucleic acid and collagens. The authors suggested that the nucleic acid-associated signal may be related to gene activation in the setting of chronic immune activation, while the collagen signal may be related to its turnover in the setting of respiratory damage. Biomarker analysis between CVID subgroups revealed decreased intensity in wavenumbers 1528 cm^−1^ in CVID+ patients, though the implication of this signal requires further investigation.

## Markers of Host-Microbial Dysregulation

### Mucosal Immunity and Microbiota

Immunoglobulins contribute to mucosal immunity and host-commensal interactions (58, 59). With the loss of antibodies, a potential role of host-commensal disruption and microbial products in inciting inflammatory disease in CVID has been the subject of a number of investigations. The initial consideration was whether there was a link between altered gut microbiota composition and inflammatory manifestations in CVID. Studies to date indicated that there was reduced gut bacterial diversity (alpha diversity) in CVID, especially in those with the CVID+ phenotype (51, 60). While the levels of various bacterial taxa had been reported to be altered in CVID, there has been no consistent taxa marking those with complications. Additionally, intermittent antibiotic use in many CVID patients remained a major confounder of concern for such an approach.

Other works have focused on the host factors that may affect this mucosal interface. One study suggested that the greater loss of mucosal IgA could be connected to gastrointestinal inflammation and intraepithelial lymphocytosis, with the activation of IFN-γ and IL-12 in this location (61). These findings were compatible with our observation of IFNγ+ ILC3 expansion in these mucosal tissues (27). In our recent study, we also sought evidence of a dysfunctional mucosal barrier in CVID by examining two biomarkers in the serum: zonulin and intestinal fatty-acid binding protein (I-FABP) (28). Zonulin is a modulator of intercellular tight junctions between epithelial cells and elevated serum zonulin levels have been linked to increased intestinal wall permeability (62). We found that serum zonulin levels were markedly elevated in CVID compared to healthy controls (mean 18.71 vs. 6.99 ng/mL, respectively, P=0.0003). I-FABP is an intestinal epithelium-specific protein that can leak into the circulation in the setting of gut barrier dysfunction, and these levels were also significantly elevated in CVID patients compared to healthy controls as well (mean 3,346 vs. 1,992 pg/mL, respectively, P=0.0006) (63). In this study, we found that serum zonulin and I-FABP levels were significantly elevated irrespective of overt enteropathy. These markers suggest that gut barrier dysfunction is frequently present in CVID – even in patients without overt gastrointestinal symptoms, and may play a role in dysregulated host-commensal balance in CVID.

With regards to the roles of microbial-derived products, early studies have focused on endotoxin as a biomarker and potential source of commensal-driven inflammation. Circulating endotoxin was reported in some studies (64, 65), but was not detected when we examined a large cohort (28) or in other publications (50, 51). In one study, circulating endotoxin was linked to reduced proliferation capacity of bacteria-specific CD4 T cells and higher expression of programmed death 1 (PD-1) on CD4+ T cells, suggesting an “exhausted” phenotype (64). However, as endotoxin was noted in CVID patients who had low serum IgG at the time of study, this marker/immune-activator may not be relevant for the majority of CVID patients who are consistently maintained on adequate immunoglobulin replacement. Other microbial products, namely their metabolites, have also been examined. Gut microbiota-derived trimethylamine N-oxide (TMAO) has previously been linked to systemic inflammation in HIV and cardiovascular diseases (66, 67). With altered gut commensals, one study found higher plasma TMAO concentration and positive correlations with select inflammatory cytokines in CVID patients (68). While neither diet nor a two-week course of rifaximin impacted plasma TMAO levels in this study, the idea of gut microbiota manipulation remains an area of active research.

### Microbial Translocation

Taking a direct approach, we recently measured bacterial 16S rDNA levels in serum samples from 92 CVID patients (28). We found that bacterial 16S rDNA was significantly elevated in CVID patients (mean 19.15, range 0.4-237.4 copies/μl) compared to healthy controls (mean 5.99, range 1.2-18.88 copies/μl, P<0.0001). High circulating bacterial DNA levels marked patients with the CVID+ phenotype and those with very few isotype-switched memory B cells (<2% of peripheral CD19+ B cells). We did not find microbial translocation to be a function of overt enteropathy or specific monogenetic defects. Bacterial DNA levels also were not correlated with serum IgG, IgA, and IgM levels – though most participants had severely reduced IgA and/or IgM in this study. 16S bacterial profiling of serum samples from 25 CVID patients revealed that the translocated bacterial rDNA predominantly belonged to various gut-associated taxa (at the phylum level: Firmicutes, Bacteroidetes, Actinobacteria, and Proteobacteria; at the family level: *Lachnospiraceae, Ruminococcaceae, Erysipelotrichaceae, Bacteroidaceae*, and *Comamonadaceae*).

Further investigation revealed that serum bacterial rDNA was closely associated with multiple disease biomarkers previously reported in CVID (28). These include a positive association with markers of monocyte activation in CVID (sCD14, as discussed above; Spearman r = 0.28, P = 0.0166). In addition, CVID patients with high serum bacterial rDNA had significantly higher serum IFN-γ levels as compared to those with low bacterial rDNA, suggesting that bacterial translocation may be a specific source of the IFN signature long observed in CVID+ (54). We further showed that circulating bacterial DNA was bioactive as it could induce significant IFN-γ secretion from the peripheral blood mononuclear cells of CVID patients, particularly those with known inflammatory conditions (P<0.0001, compared to CVID patients without inflammatory diseases). These new discoveries tie together a number of known features of immune dysregulation in CVID+ and hopefully lead to additional understanding of the pathogenesis of this complex phenotype.

## Conclusion

While CVID is traditionally classified among B-cell immune deficiencies, immunologic and genetic studies over the years have shown that it is, in many ways, a combined immune defect with additional innate and adaptive abnormalities. This has become obvious as the spectrum of autoimmune/inflammatory complications is increasing described in a major portion of the patients. As shown here, the ongoing investigations of relevant biomarkers have been useful in understanding this heterogeneous disorder and revealing its pathogenic complexity. In addition, select markers can be utilized in the clinics to identify patients at risk for additional complications and following complex conditions. Many recent works are focusing on commensal bacteria as a potential source of immune stimulation in CVID, and the presence of microbial translocation, as directly probed *via* circulating bacteria DNA, may provide a common thread among the known abnormalities of mucosal barriers, innate immunity, and adaptive immunity that marked CVID. The ongoing search of relevant biomarkers in CVID will likely reveal further shared and heterogeneous features of this disorder, and provide additional insights for clinical management, pathogenesis, and potentially new therapeutics.

## Author Contributions

HH and CC-R conceived the study, collected data, and drafted manuscript. CC-R provided critical revisions of the manuscript and final approval of the version to be published. All authors contributed to the article and approved the submitted version.

## Funding

This work was partially supported by the National Institutes of Health and National Institute of Allergy and Infectious Diseases grants AI-061093, and AI-08603.

## Conflict of Interest

The authors declare that the research was conducted in the absence of any commercial or financial relationships that could be construed as a potential conflict of interest.

## Publisher’s Note

All claims expressed in this article are solely those of the authors and do not necessarily represent those of their affiliated organizations, or those of the publisher, the editors and the reviewers. Any product that may be evaluated in this article, or claim that may be made by its manufacturer, is not guaranteed or endorsed by the publisher.
